# Lung cancer masquerading in the background of bronchiectasis: a report of two cases and literature review

**DOI:** 10.3389/fonc.2026.1751661

**Published:** 2026-03-09

**Authors:** Jian Guo, Xiaoqian Shi, Wei Zhang, Xuewei Zhao, Xiaohui Lv, Bing Li

**Affiliations:** 1 Department of Respiratory and Critical Care Medicine, Shanghai Fourth People’s Hospital, Tongji University School of Medicine, Shanghai, China; 2 Department of Pathology, Shanghai Fourth People’s Hospital, Tongji University School of Medicine, Shanghai, China; 3 Department of Thoracic Surgery, Shanghai Fourth People’s Hospital, Tongji University School of Medicine, Shanghai, China

**Keywords:** bronchiectasis, chronic airway inflammation, lung cancer, PET-CT, tumor biomarkers

## Abstract

We report two cases of lung cancer that were surgically resected from patients with underlying bronchiectasis. These cases are unique because the tumors were diagnosed neither through elevated tumor markers, hypermetabolism on PET-CT, nor the presence of typical malignant imaging features. Instead, they were incidentally identified through histopathological examination following lobectomy performed for recurrent bronchiectasis-related infections. We aim to enhance clinicians’ vigilance toward occult malignancies in patients with bronchiectasis by summarizing the treatment course of such concealed tumors. Bronchiectasis maybe associated with lung cancer. Implementing a regular imaging surveillance maybe necessary for these patients. Furthermore, timely surgical intervention should be considered when clinical or radiological findings raise suspicion of malignancy.

## Introduction

Bronchiectasis constitutes a clinical syndrome characterized by abnormal thickening and dilation of the bronchial walls visible on chest CT, accompanied by persistent cough and sputum production, recurrent infections, and intermittent acute exacerbations. It may present both as a distinct disease entity and as a manifestation of various underlying conditions—particularly chronic obstructive pulmonary disease (COPD), asthma, traction bronchiectasis associated with interstitial lung disease (ILD), or post-infectious pulmonary parenchymal destruction (1, 2). The persistent chronic inflammatory microenvironment—characterized by abundant immunosuppressive cells (including M2 macrophages, Tregs, etc.) and their associated cytokines, coupled with recurrent injury-repair cycles in the airway epithelium—constitutes a critical pathological foundation for pulmonary malignant transformation (3, 4). Current researches have established that bronchiectasis hold an elevated risk of lung cancer compared to general population. A recent systematic review demonstrated that the incidence of lung cancer in non-cystic fibrosis bronchiectasis (NCFB) ranges from 0.93% to 8.0%, with an incidence rate of approximately 3.96 per 1000 person-years. This risk is particularly significant in elderly, male, and smoking populations. The overall lung cancer risk in NCFB patients is significantly increased, showing adjusted hazard ratios (aHR) between 1.22 and 2.4 across different studies when compared to non-bronchiectasis controls, with adenocarcinoma being the most frequently reported histological subtype (5–10). However, early differentiation between bronchiectasis and occult malignancy sometimes maybe difficult. Current screening for early lung cancer primarily relies on characteristic CT findings (including spiculation, lobulation, and pleural retraction), abnormal hypermetabolism on PET-CT, or elevated tumor biomarkers (11). Particularly when tumors are concealed within dilated airways and chronic inflammatory backgrounds, the sensitivity of these screening modalities may be compromised, as the radiological features of malignancy can be obscured by structurally abnormal lung structures. This report presents two cases of lung cancer concealed within the context of bronchiectasis. Both patients underwent surgical intervention for recurrent infections, with the diagnosis of malignancy being established postoperatively as an unexpected finding, rather than being identified preoperatively. We aim to enhance physicians’ awareness of bronchiectasis maye associated with lung cancer development by sharing our clinical experience and reviewing relevant literature, while also advocating for strengthened follow-up management protocols in this patient population.

## Case presentation

We report two female patients, aged 60 and 63 years, both with no smoking history. The baseline characteristics, infection-related laboratory parameters, tumor markers, and imaging findings of these two patients are listed in **Table 1**.

**Table 1:** Patient demographics, laboratory and radiological results.

Clinical Characteristics	Case 1	Case 2
General information		
age, years	60	65
sex	female	female
smoking history	no	no
toxicant exposure	no	no
past history	breast cancer	lung cancer
Infectious agents		
T-SPOT, pg/ml	4.32	3.98
G test, pg/ml	<10	<6
GM test	<0.1	<0.1
Tumor biomarkers		
CEA, ng/ml	0.58	4.29
NSE, ng/ml	10.57	14
SCC, ng/ml	0.71	0.57
Cyc211, ng/ml	3.83	1.68
CA125, U/ml	4.78	9.38
Images		
enhanced CT	No enhancement	No enhancement
PET-CT	SUVmax 2.2	SUVmax 0.7

*Case 1 T -SPOT, T-cell Spot Forming Assay for Tuberculosis; G-Test, (1,3)-β-D-Glucan Test; GM test, Galactomannan Test; CEA, Carcinoembryonic Antigen; NSE, Neuron-Specific Enolase; SCC, Squamous Cell Carcinoma Antigen; CYFRA 21-1, Cytokeratin Fragment 211; CA125, Cancer Antigen 125; CT, Computed Tomography; PET-CT, Positron Emission Tomography - Computed Tomography.

### Case 1 presentation

**Figure 1:**
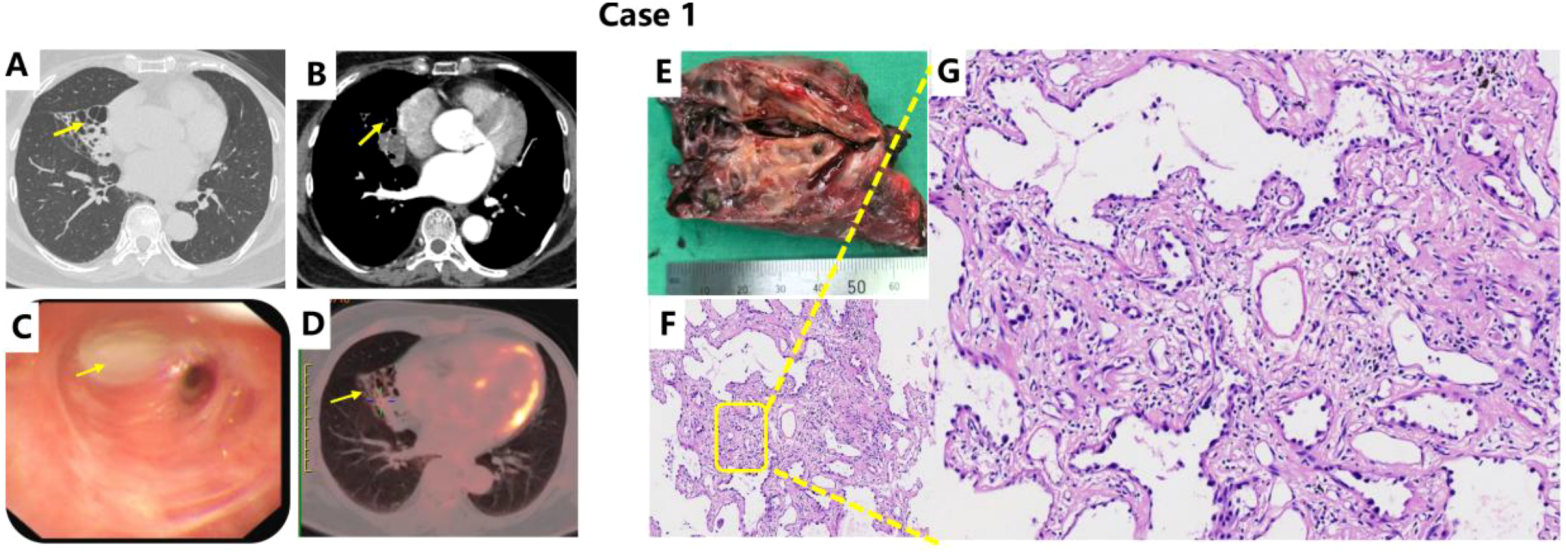
Examinations of Case 1: Contrast-enhanced chest CT, PET-CT, and Pathology. Case 1: Chest CT demonstrated bronchiectasis in the right middle lobe bronchus. However, no malignant features were observed on contrast-enhanced CT or PET-CT [**(A, B, D)**, yellow arrows]. Bronchoscopy revealed yellowish purulent sputum at the orifice of the right middle lobe bronchus, with smooth surrounding mucosa, no neoplastic lesions, and uniform narrowing of the lumen [**(C)**, yellow arrow]. **(E)** shows the postoperative specimen, with the white circle indicating the abnormally dilated bronchi; no distinct nodular or mass-like lesions were identified macroscopically. **(F)** presents the pathological section (×100, H&E staining), while **(G)** (×400, H&E staining) reveals lepidic growth of alveolar epithelium, mild thickening of alveolar walls, slight cellular atypia, and continuous monolayer proliferation of alveolar epithelium. Immunohistochemical staining was positive for TTF-1 and Napsin A, consistent with adenocarcinoma *in situ* (AIS).

(Radiological features and pathology examinations were demonstrated in **Figure 1**)

Patient case 1 was admitted to the Respiratory Department on July 14, 2025, due to persistent cough, sputum production, fever and diagnosed with bronchiectasis for many years. Chest CT performed in June 2024 revealed cylindrical bronchiectasis with thickened walls in the medial and lateral segments of the right middle lobe, accompanied by significant air trapping and a solid nodule at the distal end. The dilated bronchus exhibited a triangular configuration with the base oriented toward the hilum and well-defined margins. The patient reported intermittent respiratory symptoms and had received irregular antibiotic treatment. A follow-up contrast-enhanced CT in July 2025 demonstrated progressive bronchiectasis with destructive changes in the right middle lobe. She underwent breast cancer surgery in 2021. Postoperative pathology confirmed ductal carcinoma *in situ*, with a hormone receptor-positive (endocrine) molecular subtype. Adjuvant endocrine therapy was recommended by the breast surgeon; however, she discontinued treatment after approximately one week due to intolerable adverse effects. No chemotherapy or other adjuvant therapies were administered. Physical examination on admission showed SpO_2_ 98% on room air, stable respiration, coarse breath sounds bilaterally without audible rales. After admission, comprehensive infection screening was performed alongside empirical antibiotic therapy. Due to unsatisfactory symptomatic improvement and recurrent infections, thoracic surgery consultation was obtained. On July 21, 2025, the patient underwent video-assisted thoracoscopic right middle lobectomy under general anesthesia. Histopathological examination of the resected specimen revealed adenocarcinoma *in situ* within the dilated airways.

### Case 2 presentation

**Figure 2:**
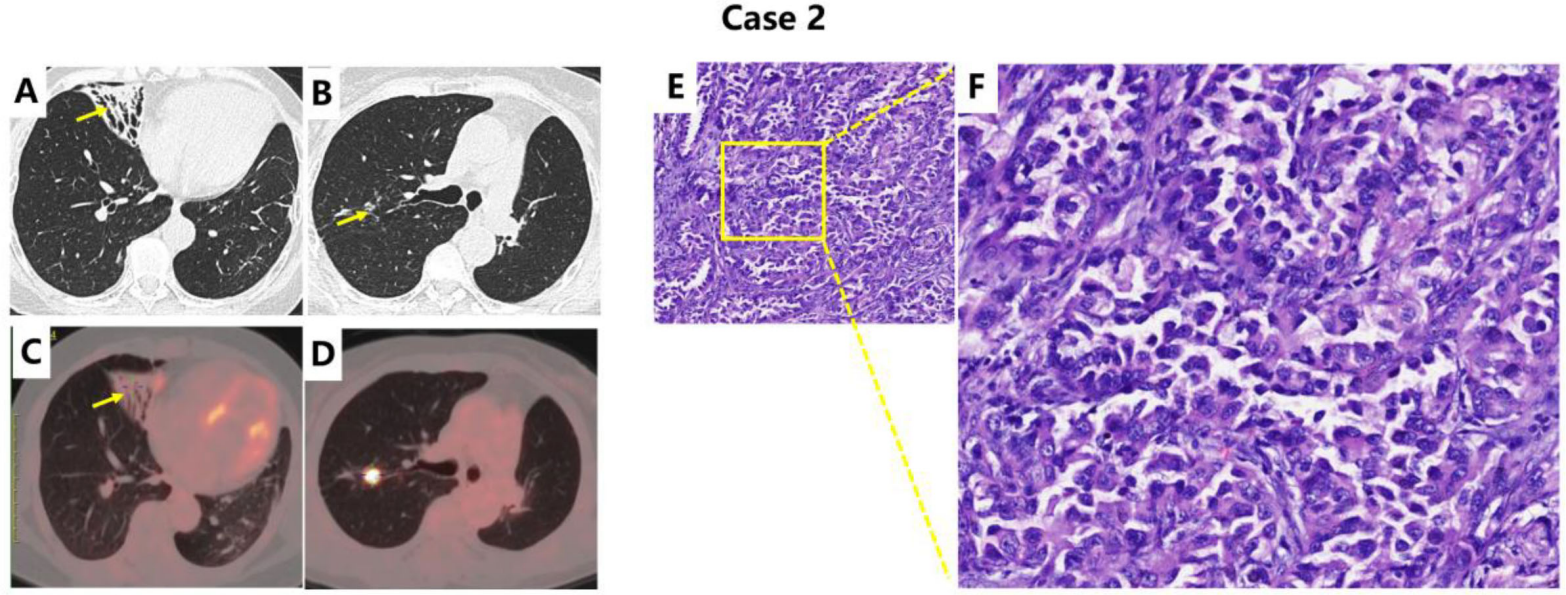
Examinations of Case 2: Contrast-enhanced chest CT, PET-CT, and Pathology. Case 2: Chest CT revealed bronchiectasis in the right middle lobe. [**(A, C)**, yellow arrows]. **(B, D)** show the high-risk nodule targeted for preoperative resection. **(E, F)** demonstrate postoperative pathological section (×100, H&E staining), while **(F)** (×400, H&E staining) demonstrates lepidic growth pattern with focal acinar components. Microscopic examination revealed typical malignant characteristics including hyperchromatic nuclei, nuclear pleomorphism, increased nuclear-to-cytoplasmic ratio, and pathological mitotic figures. Immunohistochemical staining was positive for TTF-1 and Napsin A, and negative for P40, P63, and Syn, confirming the diagnosis of adenocarcinoma. The findings are consistent with well-differentiated invasive lung adenocarcinoma.

(Radiological features and pathology examinations were demonstrated in **Figure 2**)

Patient case 2 was admitted on July 3, 2025, for a right pulmonary nodule discovered over one year earlier and also suffered from persistent cough, sputum production, fever and diagnosed with bronchiectasis for many years. She has undergone left lung adenocarcinoma surgery in 2022. The postoperative pathological stage was pT1N0M0 (Stage IA). No adjuvant chemotherapy or radiotherapy was administered. Although driver gene testing revealed an EGFR exon 19 deletion mutation, the patient did not receive targeted therapy and maintained regular follow-up, during which the right lung nodule was identified. Chest CT revealed progressive enlargement of the right solitary nodule, accompanied by cylindrical bronchiectasis in the medial segment of the right middle lobe, exhibiting bronchial wall thickening, significant air trapping, and a triangular configuration with the base oriented toward the lung periphery and well-defined margins. Physical examination on admission showed SpO_2_ 99% on room air, no cyanosis, and coarse breath sounds bilaterally without audible rales. The patient case 2 underwent a wedge resection of the right middle and lower lobes via video-assisted thoracoscopic surgery (VATS) under general anesthesia. This surgical approach was chosen because the preoperatively suspected malignant nodule was located in the lateral basal segment of the right lower lobe. Intraoperative exploration revealed bronchiectasis, structural destruction, and functional impairment in the right middle lobe, which was therefore resected concomitantly. The primary limitations of this procedure are the potential inability to achieve complete tumor removal and address metastatic lymph nodes, thereby increasing the risk of local recurrence. Postoperative pathological examination revealed that the preoperatively suspected nodule was inflammatory cell infiltration. However, an adenocarcinoma was incidentally discovered within the resected bronchiectatic tissue, measuring 2.8 cm in diameter with airway spread and no lymph node metastasis (pT1cN0M0, Stage IA3, driver gene mutation-negative). Due to the presence of high-risk factors for recurrence postoperatively, the patient received four cycles of adjuvant chemotherapy with pemetrexed plus cisplatin. Subsequent regular follow-up has shown the patient to be in stable condition with no evidence of tumor recurrence.

### Follow up and outcomes

Both patients have maintained favorable postoperative recoveries with complete resolution of cough and sputum production. They expressed satisfaction with the therapeutic outcomes and gratitude for the incidental detection and successful management of the tumors.

## Discussion

We report two cases of lung adenocarcinoma that developed covertly under a background of bronchiectasis and were unexpectedly confirmed by postoperative pathology. Both patients underwent surgical treatment for bronchiectasis with recurrent infections, with routine preoperative evaluations failing to indicate the presence of malignancy. The atypical presentation of such cases highlights the diagnostic challenges in detecting malignant tumors underlying structural lung diseases.

Bronchiectasis is a global disease with significant heterogeneities in prevalence across different countries and ethnic groups. Reported prevalences range from approximately 94.8 to 566 per 100,000 population. In China, it has been estimated that approximately 1.5% of women and 1.1% of men in the general population have physician-diagnosed bronchiectasis, representing a total affected population exceeding 15 million individuals (12–15).Given this substantial population base, even a low incidence rate of bronchiectasis-associated lung cancer could translate into a considerable absolute disease burden. Indeed, multiple recent clinical studies have confirmed a significantly higher incidence of lung cancer among patients with bronchiectasis compared to non-bronchiectasis populations (2.1 vs. 0.7 per 1000 person-years). After adjusting for potential confounders, the adjusted hazard ratio (aHR) was 1.22. This elevated risk remained statistically significant regardless of smoking status, establishing bronchiectasis as an independent risk factor for lung cancer (aHR 1.28 for never-smokers; aHR 1.26 for ever-smokers) (8). Another study from Taiwan revealed even more striking findings: the incidence of lung cancer was significantly higher in patients with bronchiectasis compared to the general population (4.58 vs. 2.02 per 1000 person-years), with an adjusted hazard ratio (aHR) of 2.36. Sex-stratified analysis demonstrated hazard ratios of 2.41 for female and 2.33 for male bronchiectasis patients. Furthermore, lung cancer incidence showed an age-dependent increase in both groups (9). However, intriguingly, another study suggested that male bronchiectasis patients might be more vulnerable to developing lung cancer. Kim et al., utilizing a large nationwide Korean database to evaluate risk factors for lung cancer in bronchiectasis subjects, identified male sex, overweight, current smoking, rural residence, and comorbid COPD as independent risk factors for lung cancer development in this patient population (16). It is noteworthy that this association may stem from shared risk factors such as smoking, chronic infection, or environmental pollutants, or from reverse causation whereby occult lung cancer leads to post-obstructive bronchiectasis. These findings therefore warrant cautious clinical interpretation, and the precise causal mechanisms require further investigation.The hypothesis has been advanced that the increased incidence of lung cancer in bronchiectasis may be attributed to the persistent chronic inflammation-injury-repair cycle, where long-standing inflammation establishes a critical tumor-promoting microenvironment (17). Historically, research on bronchiectasis has predominantly focused on bacterial respiratory infections. However, optimizing antimicrobial strategies alone has proven insufficient for achieving breakthrough therapeutic advances in this field. A recent review article published in *The Lancet Respiratory Medicine* has underscored the crucial role of inflammatory mechanisms in patients with bronchiectasis (18). Furthermore, considering the interplay between inflammation and carcinogenesis, contemporary research has unequivocally established the relationship between inflammation and cancer. Both infection-induced inflammation and immune dysregulation/autoimmunity-associated inflammation have been demonstrated to promote cancer development (19, 20).

CT serves as a crucial modality for diagnosing and evaluating early-stage tumors; however, its diagnostic specificity and sensitivity may be compromised in patients with underlying structural lung disease. The coexistence of bronchial dilation, wall thickening, peribronchial inflammation, and mucus plugs collectively impedes accurate detection of occult lesions. Serum tumor markers (e.g., CEA, SCC, NSE) exhibit inherently limited sensitivity for early-stage lung cancer and cannot reliably serve as diagnostic criteria for malignancy. While PET-CT aids in characterizing pulmonary nodules, its interpretation in bronchiectatic regions—particularly during active infection—requires careful analysis of SUV values alongside other clinical evidence due to potentially confounding metabolic activity. We analyze that the absence of uptake on PET-CT in these two patients may be attributed to the small size of the tumor and/or the high background FDG avidity from inflammatory activity in the bronchiectatic region, which could mask abnormal metabolic signals from the tumor itself. This attenuation of diagnostic efficacy may lead to significant underdiagnosis in clinical practice.

Based on the serendipitous findings in our two cases and corroborating observational evidence, bronchiectasis should not be oversimplified as a purely infectious disorder. Accumulating data indicate that this patient population may harbor an increased risk for lung cancer compared to the general public, justifying heightened clinical awareness. A critical caveat is that both reported patients had previous malignancies, yet neither reported a family history of cancer nor prior exposure to radiotherapy or chemotherapy. The exact influence of a prior cancer—especially one of distinct histology or driver gene profile—on the subsequent emergence of a new neoplasm within bronchiectatic airways remains to be elucidated. Moreover, whether individuals with a history of adjuvant radiotherapy or chemotherapy bear a greater risk of secondary primary tumors relative to those with bronchiectasis alone or healthy controls necessitates further empirical support. In patients with bronchiectasis, the detection of suspicious imaging abnormalities or a strong clinical suspicion of malignancy should prompt the judicious and timely employment of invasive diagnostic modalities. Surgical resection holds particular merit in this context, serving a dual purpose: it establishes a conclusive pathological diagnosis while simultaneously offering a potentially curative intervention, as illustrated by the two cases described herein.While the observations from this report are illuminating, their broader implications are constrained by the limited cohort size, resulting in inadequate statistical power and restricted external validity. Future investigations should focus on implementing large-scale, multi-center, prospective cohort studies with extended follow-up durations to precisely determine the incidence and attributable risk of lung cancer in individuals with bronchiectasis. Concurrently, the development of artificial intelligence models that synthesize clinical, radiomic, microbiomic, and molecular biomarker data holds promise for refining predictive accuracy in early lung cancer detection and guiding tailored surveillance approaches for this at-risk population.

## Conclusions

The two representative cases in this report demonstrate that lung cancer may arise and progress in an exceptionally occult manner among patients with bronchiectasis. Its radiological, clinical, and laboratory manifestations can be masked by concurrent infections and structural alterations. While it remains to be established through larger-scale clinical studies whether bronchiectasis itself constitutes associated with lung cancer development, our findings serve to alert clinicians to this potential association. Particular attention is warranted for patients with bronchiectasis who experience persistent, recurrent infections poorly responsive to medical therapy, or those with documented high-risk co-factors such as heavy smoking history or prior treatment with anti-cancer or immunosuppressive agents. Implementing regular CT surveillance, conducting longitudinal comparisons of imaging studies, and pursuing timely, aggressive diagnostic interventions for suspicious lesions can facilitate the early detection and prompt management of occult lung malignancies in this setting.

## Data Availability

The original contributions presented in the study are included in the article/**Supplementary Material**. Further inquiries can be directed to the corresponding author.
